# Editorial: Liquid Biopsy as a Tool for Precision Oncology: New Challenges to Assess Clinical Response

**DOI:** 10.3389/fphar.2020.598261

**Published:** 2020-10-07

**Authors:** Gloria Ravegnini, Marzia Del Re, Ambra A. Grolla, Ron H. N. van Schaik, Sabrina Angelini

**Affiliations:** ^1^ Department of Pharmacy and Biotechnology, University of Bologna, Bologna, Italy; ^2^ Unit of Clinical Pharmacology and Pharmacogenetics, Department of Clinical and Experimental Medicine, University of Pisa, Pisa, Italy; ^3^ Department of Pharmaceutical Sciences, University of Piemonte Orientale, Novara, Italy; ^4^ Department of Clinical Chemistry, Erasmus MC, Rotterdam, Netherlands

**Keywords:** liquid biopsy, precision oncology, ctDNA (circulating tumor DNA), Circulating molecular marker, personalized medicine

The Research Topic “Liquid Biopsy as a Tool for Precision Oncology: New Challenges to Assess Clinical Response” includes eight papers with more than 50 authors contributing as experts in the field. The Research Topic has the aim to reflect the state of the art on this emerging technology, which is revolutionizing the clinical approach in oncology.

Despite the numerous progresses in early diagnosis and in target therapies, cancer remains the second leading cause of death worldwide. Tumor molecular characterization plays a key role in choosing the right treatment among the armamentarium available to the oncologist. Unfortunately, with the traditional tissue biopsy in some cases analysis of tumor is not feasible due to insufficient or poor quality material (Siravegna et al., 2019). Liquid biopsy has started to be considered a new standard of care for oncological patients mainly after the FDA approval on 2016 of the first blood-based test, to detect EGFR mutations for selecting patients who may benefit from the target therapy (Torres et al, 2020). Indeed, liquid biopsy has several advantages, including its minimal invasiveness and highly repeatability over the time, which potentially guarantees a dynamic picture of the tumor and the chance to monitor pharmacological responses. This last characteristic makes liquid biopsy particularly attractive within the oncological context. Indeed, to date, diagnosis and clinical monitoring have been the two major applications of liquid biopsy, and are well described in our Research Topic.

Kamatham et al., who used circulating tumor DNA (ctDNA) to identify, microsatellite instability status in a pancreatic cancer patient. This led to switch the therapy from chemo to immuno-therapy, with an excellent response. A different case report is presented by Nagaya et al., which uses circulating tumor cell (CTC) analysis to select the right treatment in castration-resistant prostate cancer (CRPC) patients. In this article, the authors evaluated AR-V7 expression in CTC in serial blood tests; based on the results, abiraterone was selected as re-challenge in the setting of post-chemo androgen-targeted-therapy.

Another example of a clinical application of liquid biopsy is presented by Dalle Fratte et al., present a case report of a patient with KIT/PDGFRA wild-type gastrointestinal stromal tumor (GIST). The patient was resistant to the standard treatment imatinib, even after increasing the dose. By using an NGS based approach, the authors analyzed circulating free DNA (cfDNA) to investigate somatic changes responsible for imatinib resistance, and identified a sharp increase in the allele frequency of a TP53 mutation, responsible for a protein loss of function, never described before. The same TP53 mutation was retrospectively identified in the primary tumor and in the metastatic hepatic lesion, suggesting a rapid clonal selection of the mutation during tumor progression. Besides these case reports, that highlight the potential of liquid biopsy in different cancer types with no previous clinical indications, two contributions describe the application of liquid biopsy in a high number of patients. Buderath et al. evaluated levels of soluble PD-L1 and PD-L2 in sera of 83 primary epithelial ovarian cancer (EOC) patients and related the results with the presence of CTCs, clinical characteristics and with PFS and OS. The results showed that sPD-L1 and sPD-L2 could be used as complementary biomarkers reﬂecting clinical status, treatment response and disease outcome of EOC patients. In particular, sPD-L1 may facilitate the identiﬁcation of high-risk patients with unfavorable disease outcomes despite platinum-sensitivity arguing for additional therapeutic approaches. Pazdirek et al. investigated the potential of ctDNA as blood-based biomarker in patients with locally advanced rectal cancer (LARC) undergoing neoadjuvant chemoradiotherapy (NCRT) prior to surgery. The study included 36 LARC patients undergoing NCRT followed by surgical treatment. Somatic mutations were characterized in tissue biopsies and ctDNA from plasma samples prior to therapy and at the end of the first week of treatment (plasma only). The analysis revealed a ctDNA reduction within the 1^st^ week of therapy, not associated with the clinical response determined. The rapid ctDNA disappearance, unrelated with the therapy outcome, suggests the need for further studies to better understand its clinical significance in LARC patients within the neoadjuvant setting.

Finally, three reviews illustrate the state of the art about the scientific progress in liquid biopsy in different cancer types, highlighting novel aspects. Cavallari et al. provide a critical summary on the potential applications and its pitfalls of circulating molecules in Malignant Pleural Mesothelioma (MPM), an aggressive tumor linked to asbestos exposure. This is often diagnosed at an advanced stage with consequent poor prognosis. MPM diagnosis relies on pleural biopsies, therefore, the development of circulating biomarkers for early diagnosis is of great importance. In particular, the identification of robust non-invasive tests for the screening and risk assessment of asbestos-exposed subjects is still an important unmet clinical need. In this context, the authors reviewed the recent literature with the aim to identify novel blood-based circulating biomarkers for the early diagnosis and prognostic stratification of MPM patients. Despite some other interesting potential biomarkers, including miRNAs, CTCs, and ctDNA, validation in large prospective studies is still awaited.

Chennakrishnaiah et al. focus their attention on the so-called leukobiopsy. Recently, it has been reported that white blood cells (WBCs) may represent a sort of reservoir of circulating oncogenic DNA. Indeed, circulating WBCs, especially neutrophils, may contain high levels of oncogenic DNA, exceeding the amount of normally recovered from soluble fractions of plasma, circulating extracellular vesicles, platelets, and others. In some settings, the WBC content of cancer specific DNA (csDNA) may represent an important source of csDNA contained in serum or in plasma. Another thrilling topic is presented by Tieng et al., who describes the importance of CTC single-cell transcriptomics analyses in colorectal cancer (CRC). Single-cell RNA sequencing (scRNA-seq) is emerging as a breakthrough technology which provides the potential to dissect the different cellular populations of cancers and possibly the intra tumor heterogeneity (ITH). An important point raised by the authors is the role of CTC in CRC metastasis and their characterization through scRNA-seq.

In conclusion, the “Liquid Biopsy as a Tool for Precision Oncology: New Challenges to Assess Clinical Response” Research Topic highlights the importance of circulating molecules as a new tool to achieve personalized therapy. In the last decade, many progresses have been done, but at the same time, many clinical aspects remain to be elucidated. In this context, the papers enclosed in this topic offer the chance to generate a collaborative discussion, contributing to the future direction of liquid biopsy.

## Author Contributions

GR, MR, AG, RS, and SA wrote the editorial and revised the final drafts. All authors contributed to the article and approved the submitted version.

## Conflict of Interest

The authors declare that the research was conducted in the absence of any commercial or financial relationships that could be construed as a potential conflict of interest.
